# Comparative study between bone marrow mononuclear fraction and mesenchymal stem cells treatment in sensorimotor recovery after focal cortical ablation in rats

**DOI:** 10.1186/1744-9081-8-58

**Published:** 2012-12-13

**Authors:** Helder Teixeira de Freitas, Viviane Gomes da Silva, Arthur Giraldi-Guimarães

**Affiliations:** 1 Laboratório de Biologia Celular e Tecidual - Centro de Biociências e Biotecnologia, Universidade Estadual do Norte Fluminense Darcy Ribeiro (UENF), Av. Alberto Lamego, 2000 - Parque Califórnia, Campos dos Goytacazes, RJ, Brazil; 2 Setor de Apoio em Biologia Celular da Unidade de Experimentação Animal - sala 094 do Hospital Veterinário - UENF, Av. Alberto Lamego, 2000 - Parque Califórnia, Campos dos Goytacazes, RJ, CEP: 28013-602, Brazil

**Keywords:** Motor cortex, Cell therapy, Stem cell, Functional recovery, Structural plasticity

## Abstract

**Background:**

Different models of cortical lesion lead to different effects on plasticity of connections and loss of function. In opposition to ischemia, cortical lesion made by ablation does not induce significant adaptive plasticity of corticocortical and corticostriatal projections and leads to functional alterations other than those observed after ischemia. We have demonstrated sensorimotor recovery after treatment with bone marrow-derived mesenchymal stem cells (MSCs) or bone marrow mononuclear cells (BMMCs) in a model of focal cortical ischemia. Here, we extended this analysis evaluating the effect of these cells on sensorimotor recovery after focal cortical ablation, reproducing the same size and location of previous ischemic lesion.

**Findings:**

Focal cerebral aspiration of the six cortical layers in left frontoparietal cortex was performed in male Wistar rats. One day later, MSCs or BMMCs were administrated (i.v.) in the ablated animals. Vehicle was administrated in the control group. Sensorimotor tests were performed before and after injury followed by i.v. injection. The monitoring of functional recovery was performed weekly during three post-ablation months. The results showed significant sensorimotor recovery with both treatments, whereas control groups had no recovery. Moreover, both cell types induced the same level of recovery.

**Conclusions:**

Bone marrow cells showed therapeutic efficacy in a model of brain injury known to promote low structural plasticity. Thus, the results support the idea of BMMCs as better candidates to treat acute CNS injuries than MSCs, since they have the same therapeutic potential, but its obtainment for autologous transplantation has been shown to be faster and easier.

## Background

Several reports have suggested a promising potential of bone marrow-derived cells to treat brain ischemia [1]. Most studies have used bone marrow-derived mesenchymal stem cells (MSCs) [2]. However, bone marrow mononuclear cells (BMMCs) has also been shown to be beneficial [3,4]. Moreover, MSCs and BMMCs seem to have similar potential to promote tissue protection and functional recovery [5-7].

Studies with sensorimotor cortical ischemia revealed ischemia-induced structural plasticity [8-10]. Surprisingly, it does not seem to occur when lesion is made by ablation [8-11]. Functional evaluation showed differences in the sensorimotor deficits among these two forms of cortical lesion [12].

We have shown that MSCs and BMMCs induce the same level of sensorimotor recovery after sensorimotor cortical ischemia [3,5]. Here, we extended this analysis, evaluating whether MSCs and BMMCs are able to promote sensorimotor recovery after sensorimotor cortical ablation, a model known for not being able to induce significant structural plasticity [9,10].

## Methods

Adult male Wistar rats (2–4 months old) weighing 250 to 350 g were homogeneously distributed between experimental groups. Experimental procedures were approved by the Animal Ethics Committee of our institution.

Ablation was performed by aspiration, as previously described [13]. Briefly, after anesthesia with ketamine hydrochloride (90 mg/kg, i.p.) and xylazine hydrochloride (10 mg/kg, i.p.), the left frontoparietal cortex (+2 to −6 mm A.P. from the bregma) was exposed and aspirated with a pipette tip (1 ml) attached to a vacuum pump. A piece of collagen haemostatic sponge was put inside the lesion, the skin was sutured, and the animals were returned after recovery from anesthesia.

Four ablated animals were sacrificed 24 h after ablation. Brains were removed and sectioned in the coronal plane at 2 mm of thickness. The slices were immersed for 30 min into 2% 2,3,5-Triphenyltetrazolium chloride (TTC) solution at 37°C. Digital images were captured with a camera coupled to a dissecting microscope and a PC computer.

Bone marrow was harvested aseptically from tibias and femurs of naive donor rats (2–4 months old), as previously described [3]. BMMCs were collected and washed with phosphate-buffered saline (PBS). After cell counting, they were resuspended in PBS, and the final concentration was approximately 3 x 10^7^ BMMCs/500 μl.

The protocol for MSCs cultivation used in this work has been already shown to be effective to obtain enriched cultures [5]. Briefly, BMMCs were plated in flasks with DMEM-F12 supplemented with 10% fetal bovine serum (GIBCO BRL). After cells from each flask had been reached the confluence, they were harvested and replated in two new flasks for expansion. After the third replating, cells were harvested and suspended in PBS, and the final concentration was approximately 3 x 10^6^ MSCs/500 μl.

Twenty four hours after ablation, animals were anesthetized and injected through the left jugular vein with MSCs, BMMCs or vehicle (PBS). Experimental groups are explained in Table 1.

**Table 1:** Experimental groups

**Group name**	**Treatment**	**Total number of animals**	**Submitted to cylinder test**	**Submitted to adhesive test**
MSCs group	3 x 10^6^ MSCs in 500 μl	8	8	7
BMMCs group	3 x 10^7^ BMMCs in 500 μl	9	9	9
Control group	500 μl of PBS	18	13	18

Blinded investigators performed the analyses to avoid bias. Animals of all experimental groups were tested one day before the ablation and at post-ablation day (PAD) 2, and then weekly until PAD 91. The pre-ablation day was plotted in graphs as PAD 0. Two functional tests were used, as previously described to assess sensorimotor function after similar cortical lesion by ischemia [3,5]. Briefly: 1- Forelimb use asymmetry (cylinder) test: the trial consisted of placing the animal inside a glass cylinder and counting supports in the wall with ipsilateral (to the lesion), contralateral or with both forelimbs. Animals with asymmetry score higher than 15 at PAD 0 or lower than 30 at PAD 2 were discarded. 2- Adhesive removal patch test: a small round adhesive paper (13 mm diameter) was placed on the inner portion of each wrist of the animal, and percentage of contralateral preference relative to the total number of removals was calculated. Animals with contralateral preference lower than 25% at PAD 0 or higher than 0% at PAD 2 were discarded.

Repeated measures two-way ANOVA was used for comparison among groups. Tukey post-hoc test was used when interaction was significant. When interaction was not significant, data from all PADs were grouped as replicates for each animal. Means were plotted for analyses with *t* test (two groups) or ANOVA plus Tukey post-hoc test (three groups). The level of significance was always set at *p* < 0.05.

## Results

**Figure 1:**
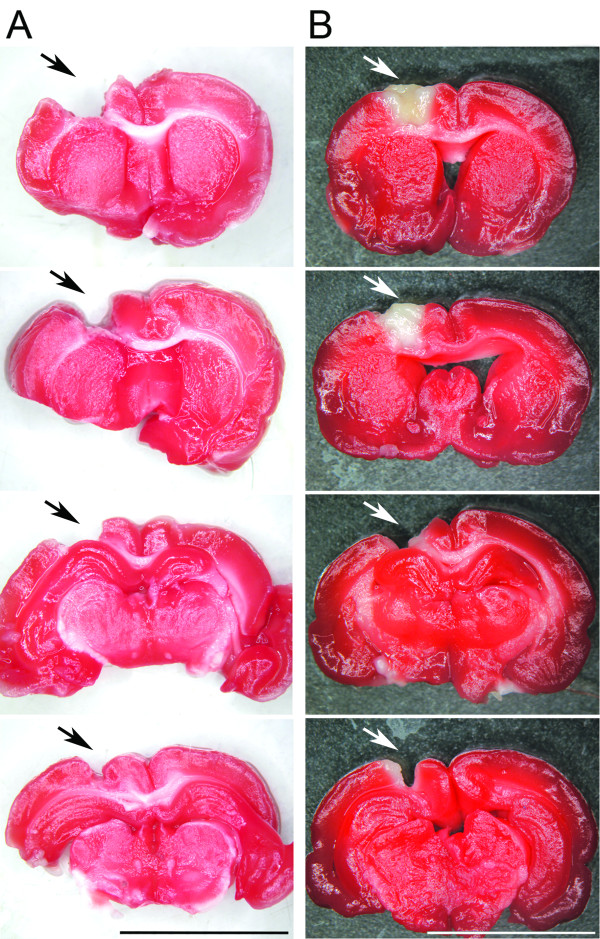
**Extension of the lesion induced by ablation.** (**A**) Sequential images of coronal brain slices of a representative ablated animal stained with TTC, showing the extension of the lesion in the anterior-posterior axis. Unilateral ablation removed the six cortical layers in the dorsal portion of the hemisphere, reaching white matter. The medial-lateral and anterior-posterior extensions of the lesion were made to reproduce the same extension of cortical lesion made by thermocoagulation in previous studies [3]. (**B**) Images captured from coronal brain slices of a thermocoagulated ischemic animal analyzed in a previous study, to illustrate the highly similar lesion extension of both protocols of injury. Calibration bars = 1 cm. In (**A**) and (**B**), from top to bottom, images were placed from most anterior to most posterior portion of the lesion. Black (**A**) and white (**B**) arrows point to the place of the lesion induced by the respective protocol of surgery.

**Figure 2:**
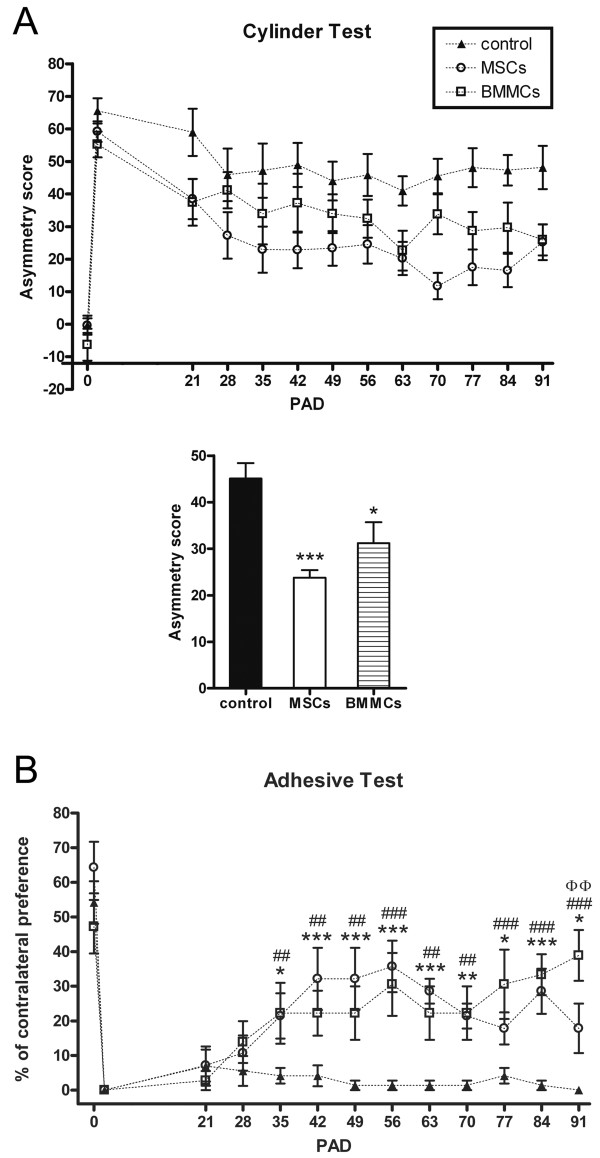
**MSCs and BMMCs promoted the same level of recovery.** (**A**) Upper graph: sensorimotor function over time in the Cylinder Test. In all groups, the greater asymmetry was observed at PAD 2. Two-way ANOVA analysis revealed no significant interaction, but significant effect of the treatment. Thus, MSCs curve and BMMCs group curve were significantly closer to normal state (PAD 0) than the control group curve. Points in the graph mean mean±SEM. Lower graph: Data from each group was grouped and analyzed by ANOVA followed by a post-hoc analysis, which showed significant recovery in MSCs and BMMCs groups, but no significant difference between them. Bars mean mean±SEM. * = *p* < 0.05, *** = *p* < 0.001; Tukey. (**B**) Sensorimotor function over time in the Adhesive Test. Legend in (**A**) is valid in (**B**). In all groups, the lower level of contralateral preference was observed at PID 2. Two-way ANOVA analysis revealed a significant interaction and post-hoc analysis showed significant recovery in MSCs and BMMCs groups from the PAD 35 onwards. However, significant difference between them was found only at PAD 91. Points in the graph mean mean±SEM. * represents comparison among MSCs and control groups, # represents the comparison among BMMCs and control groups and Φ represents the comparison among MSCs and BMMCs groups. (* = *p* < 0.05; **, ## or ΦΦ = *p* < 0.01; *** or ### = *p* < 0.001; Tukey).

After ablation by aspiration, lesions were restricted to the removed tissue, without any additional injury (Figure 1). No animal showed spontaneous rotational movement and/or abnormal curved posture toward the lesion side, which indicated the absence of striatal lesion [14,15]. Ablation mimicked the extension of cortical lesion that was induced by thermocoagulation of cortical superficial blood vessels in previous studies [3,5] (Figure 1). The lesion was effective to promote loss of sensorimotor function, since all animals showed clear asymmetry in favor of ipsilateral (unimpaired) forelimb at PAD 2 in both functional tests (Figure 2).

Analyses with sensorimotor tests revealed that MSCs and BMMCs were able to recover function. In Cylinder Test (Figure 2A), repeated measures two-way ANOVA revealed no significant interaction (F = 1.03; *p* = 0.43) and significant effects of treatment (F = 9.98; *p* < 0.001) and time (F = 18.36; *p* < 0.0001). Data of all PADs were grouped, and ANOVA showed a significant difference (F = 9.98; *p* < 0.001). Comparison between groups showed significant recovery in MSCs and BMMCs groups, but no significant difference between them (Figure 2A). In Adhesive Test, repeated measures two-way ANOVA revealed a significant interaction (F = 3.93; *p* < 0.0001). Post-hoc analysis showed significant recovery of MSCs and BMMCs groups from the PAD 35 onwards (Figure 2B). Regarding the comparison between MSCs and BMMCs groups, post-hoc analysis showed no significant difference, except at PAD 91, when the recovery induced by BMMCs was significantly higher (Figure 2B). Together, these results indicate that both treatments induced the same level of recovery.

## Discussion

The induction of sensorimotor recovery after cortical ablation by bone marrow-derived MSCs is in agreement to previous demonstrations of their efficiency to treat CNS injuries [2]. However, we used the therapeutic window of 24 h after ablation, a protocol that is only possible using donor animals. Autologous transplantation of MSCs is unfeasible after too short time window, since its enrichment and its expansion are obtained after successive replating and cultivation for weeks [16]. This delay hampers transplantation in acute or subacute phases of CNS lesions, when therapy should be more successful.

BMMCs have been suggested as an alternative for treatment of acute CNS injuries [1,3,17]. They can be obtained without cultivation and can be harvested in 1.5-6 h for autologous administration [18,19]. In fact, BMMCs seem to be only effective when administrated in the acute/subacute phase of cerebral ischemia [4,5]. We showed that BMMCs induced sensorimotor recovery with the same effectiveness of MSCs after cortical ablation. These results are in agreement to other comparative reports, which are scarce but have given support to the idea of BMMCs as an alternative to MSCs [5-7]. Moreover, we observed that the recovery promoted by BMMCs was sustained for at least three months, which is consistent with prior reports from our research group [5].

The present study deepens demonstration of therapeutic potential of bone marrow-derived cells, since we used a model of cortical lesion not yet used for cell-based therapies. Cortical ablation mimics a clinical condition of immediate removal of brain tissue, e.g., surgical removal of brain tumors. It is necessary to verify cell therapies efficacy in this debilitating condition, although its occurrence is significantly lower than stroke or traumatic brain injury (TBI). Moreover, cortical ablation model should be attractive for studies about mechanisms of action of therapies. Ablation differs from the natural inflammatory process of tissue removal that occurs after ischemia and TBI. Studies have suggested that inflammation induces lesion-induced structural plasticity, which is not observed after ablation [8,10]. Increasing evidences have shown that inflammation triggers a return to a development-like condition, more permissive to axon regeneration and lesion-induced structural remodeling [8,20]. Thus, our results were of interest, since we showed sensorimotor recovery induced by cell therapies in a model of lesion that does not cause significant structural plasticity [8-10].

Bone marrow-derived cells have been described to migrate to injured tissue and act as a local “factory” of molecules, promoting angiogenesis, neuroprotection, modulation of inflammatory response, reduction of glial scar and facilitation of axonal regeneration and plastic rewiring of neuronal connections [2,21,22]. In preliminary experiments, we have not found systemically administrated MSCs or BMMCs in the cortical lesion made by ablation (data not shown), which might suggest that bone marrow cells would act in ablation by systemic release of factors. However, further observations are needed to prove it.

Concluding, we demonstrated that MSCs and BMMCs are equally able to promote sensorimotor recovery. Thus, the study supports BMMCs as a cheaper and faster alternative for the treatment of CNS lesions, ideal for autologous transplantation in acute and subacute phases. Furthermore, these cells were able to promote recovery in a model of cortical lesion known for not being able to promote significant structural plasticity, a phenomenon believed to be essential for functional recovery. Thus, further studies are needed to explain the mechanisms underlying this recovery, verifying whether these cells are able to induce structural plasticity after ablation, and/or whether other mechanisms are lying beneath this therapeutic effect.

## Abbreviations

ANOVA: Analysis of variance; BMMCs: Bone marrow mononuclear cells; CNS: Central Nervous System; MSCs: Mesenchymal stem cells; PBS: Phosphate-buffered saline; PAD: Post-ablation day; SEM: Standard error of the mean; TBI: Traumatic brain injury.

## Competing interests

The authors declare that there are no conflicts of interest.

## Authors’ contributions

HTF and VGS performed the experiments and participated in interpreting the results. AGG designed the study, supervised execution, participated in interpreting the results, performed statistical analysis and wrote the manuscript. All authors read and approved the final manuscript.

## Authors’ information

Laboratory of Cell and Tissue Biology, Center for Biosciences and Biotechnology, State University of the Northern Rio de Janeiro, Brazil.
